# Mutations in an AP2 Transcription Factor-Like Gene Affect Internode Length and Leaf Shape in Maize

**DOI:** 10.1371/journal.pone.0037040

**Published:** 2012-05-23

**Authors:** Fukun Jiang, Mei Guo, Fang Yang, Keith Duncan, David Jackson, Antoni Rafalski, Shoucai Wang, Bailin Li

**Affiliations:** 1 National Maize Improvement Center of China, College of Agriculture and Biotechnology, China Agricultural University, Beijing, China; 2 DuPont Agricultural Biotechnology, Wilmington, Delaware, United States of America; 3 Pioneer Hi-Bred International, Johnston, Iowa, United States of America; 4 Cold Spring Harbor Laboratories, Cold Spring Harbor, New York, United States of America; University of Massachusetts Amherst, United States of America

## Abstract

**Background:**

Plant height is an important agronomic trait that affects yield and tolerance to certain abiotic stresses. Understanding the genetic control of plant height is important for elucidating the regulation of maize development and has practical implications for trait improvement in plant breeding.

**Methodology/Principal Findings:**

In this study, two independent, semi-dwarf maize EMS mutants, referred to as *dwarf & irregular leaf* (*dil1*), were isolated and confirmed to be allelic. In comparison to wild type plants, the mutant plants have shorter internodes, shorter, wider and wrinkled leaves, as well as smaller leaf angles. Cytological analysis indicated that the leaf epidermal cells and internode parenchyma cells are irregular in shape and are arranged in a more random fashion, and the mutants have disrupted leaf epidermal patterning. In addition, parenchyma cells in the *dil1* mutants are significantly smaller than those in wild-type plants. The *dil1* mutation was mapped on the long arm of chromosome 6 and a candidate gene, annotated as an AP2 transcription factor-like, was identified through positional cloning. Point mutations near exon-intron junctions were identified in both *dil1* alleles, resulting in mis-spliced variants.

**Conclusion:**

An AP2 transcription factor-like gene involved in stalk and leaf development in maize has been identified. Mutations near exon-intron junctions of the AP2 gene give mis-spliced transcript variants, which result in shorter internodes and wrinkled leaves.

## Introduction

Certain architecture characteristics of crop plants are associated with increased yields [1], [2]. Plant height, an important component of plant architecture, is highly correlated with biomass yield and significantly affects grain yield. Shorter plants are more tolerant to lodging, while more erect leaves or smaller leaf angle can lead to high planting density adaptation and yield enhancement. However, increasing demand for lignocellulosic biomass for biofuel production may lead to a shift in desirable plant architecture characteristics [3].

The application of semi-dwarf varieties in wheat and rice breeding resulted in a significant yield increase during the “Green Revolution” of the 1960s [4]. Among the genes responsible for these semi-dwarf phenotypes are the *semidwarf1* (*sd1*) gene of rice and the *Reduced height* (*Rht*) gene of wheat [5], [6], [7]. *Sd1* encodes a GA20-oxidase, an enzyme involved in the biosynthesis of gibberellic acid (GA). *Rht* encodes a DELLA transcription factor-like protein containing an SH2-like domain, and the *Rht* mutation causes a reduced response to GA. A lot of attention has been given to Dwarf 8 (D8, the maize homologue of *Rht*)and D9 (a D8 paralog) [8]. D8 contains N-terminal DELLA and VHYNP domains essential for GA-dependent degradation of proteins. The modification of the DELLA domain in D8 causes a dominant, gain of function mutation [5]. Recently, the cloning of *D8-1023*, an allele of *D8*, indicates the VHYNP domain is also important for gene function [9].

Besides the dominant mutants *D8* and *D9*, many dwarf mutants have been isolated in maize and several genes responsible for the dwarf phenotype have been cloned, all of them involved in hormone metabolism or transport. *An1* is involved in the biosynthesis of ent-kaurene, the first tetracyclic intermediate in GA biosynthetic pathway [10]. *Dwarf3* encodes a cytochrome P450 protein, which mediates an early step in the GA biosynthesis pathway [11]. The dwarf phenotype of *brachytic2* (*br2*) is caused by mutation in an ATP-binding cassette transporter of the multidrug resistant (MDR) class of P-glycoproteins (PGPs), which is involved in IAA export from intercalary meristems [12]. Recently, the classic maize dwarf mutant *na1* (*nana plant-1*) was cloned and found to encode a key enzyme in the brassinosteroid biosynthetic pathway [13].

In order to better understand the mechanism of plant height regulation, we isolated and characterized two new semi-dwarf mutants in maize. They were found to be allelic and named *dwarf & irregular leaf* (*dil1)* due to their stature (shorter internodes) and shorter, wider, and wrinkled leaves. We cloned the DIL1 gene through a map-based cloning approach. It encodes an AP2 transcription factor-like gene. Interestingly, both *dil1* alleles are defective in intron splicing, resulting in aberrant processed transcripts.

## Results

### Identification of Two Maize Mutants with Impaired Stalk and Leaf Development

**Figure 1:**
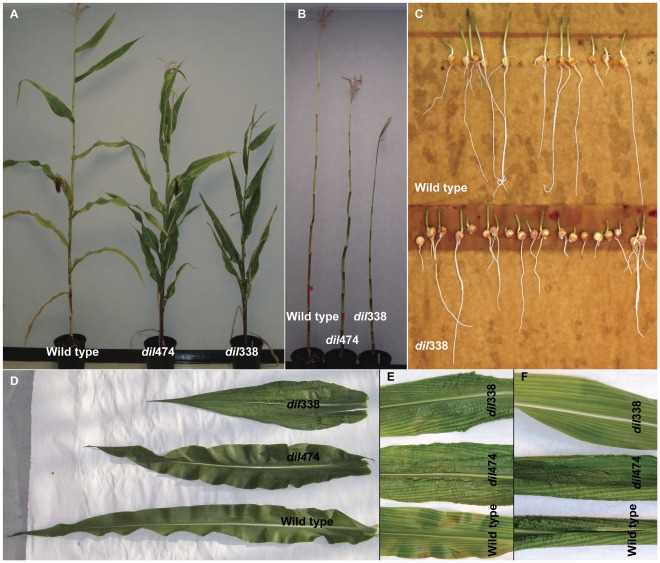
Characterization of *dil1* mutant phenotype. **A**. *dil1* mutant plants (9-weeks-old) are characterized by their reduced plant height and smaller leaf angles as compared to the wild type plants. **B**. Stalks of wild type and *dil1* mutant plants (9-weeks-old) with leaves and ears removed. **C**. Wild type (upper) and *dil*338 mutant (lower panel) seedlings six days after germination in dark. **D**. Abaxial surface of the first leaf below the ear in 9-weeks-old wild type and *dil1* mutant plants. **E** and **F**. Close look of abaxial (E) and adaxial (F) surfaces of the first leaf below the ear in 9-weeks-old wild type and *dil1* mutant plants.

Two independent semi-dwarf mutants, *dil*474 and *dil*338 with similar phenotypes were isolated from an EMS mutagenized PHN4611 population. Homozygous mutant plants have reduced plant heights due to shorter internodes (Figure 1A, 1B). Both *dil1* mutants also have more erect, shorter but wider, crinkled leaves (Figure 1A, 1D, 1E and 1F). Leaf angles, leaf length and width, internode length and plant height were measured in 12 mature plants from each genotype (homozygous wild type, *dil*338 and *dil*474) to better quantify the variations. The mutant plants are significantly different from the wild type in all the parameters measured (Table 1). In addition, the root and seedling lengths of one-week-old mutant seedlings seem to be shorter than that of wild type seedlings (Figure 1C). In summary, the *dil1* mutations result in reduced plant height and changes in leaf shape.

**Table 1:** Comparison of leaf angle, leaf length and width, internode length and plant height between wild-type and *dil1* mutant plants.

Trait	Wild type	*dil*338	*dil*474
Leaf angle: 3rd leaf above ear (degree)	48.2	28.4***	18.2****
Leaf angle: 3rd leaf below ear (degree)	36.1	28.8***	20.0****
Leaf length: 3rd leaf above ear (cm)	63.2	41.6****	39.1****
Leaf length: 3rd leaf below ear (cm)	103.3	62.7****	58.5****
Leaf width: 3rd leaf above ear (cm)	8.2	11.1****	9.3*
Leaf width: 3rd leaf below ear (cm)	7.6	10.7****	9.1**
Internode length: 3rd above ear (cm)	17.0	9.6****	11.2****
Internode length: 3rd below ear (cm)	19.3	10.0****	11.1****
Plant height (cm)	192.6	136.3****	134.4****

Means of 12 plants. Number of stars (*) indicates level of significance from Student t-test:

* 0.01≤P≤0.05,

** 0.001≤P≤0.01,

*** 0.0001≤P≤0.001,

**** P≤0.0001.

### Abnormal Leaf Epidermal and Stalk Parenchyma Cells in *dil1* Mutant Plants

**Figure 2:**
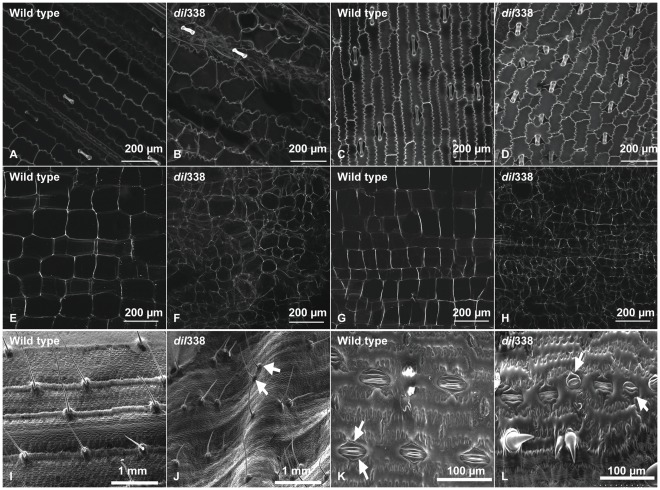
Cytological observations of *dil1* mutant. **A** and **B**: Multiphoton laser scanning micrograph (LSM) images of the adaxial surface of leaf epidermis from the 2^nd^ leaf of V3 stage wild type (WT) (A) and *dil*338 (B) seedlings. **C** and **D**: Multiphoton LSM images of the adaxial surface of leaf epidermis at post-flowering stage in WT (C) and *dil*338 (D) plants. **E** and **F**: Multiphoton LSM images of stalk parenchyma cells from third internode (from base) at post-flowering stage in WT (E) and *dil*338 (F) plants. **G** and **H**: Multiphoton LSM images of stalk parenchyma cells from ninth internode (from base) at post-flowering stage in WT (G) and *dil*338 (H) plant. **I** and **J**: The adaxial surface of WT leaf with flat blade and regularly spaced macrohairs (I), and *dil*338 with an undulating leaf surface and clustered macrohairs (J, arrows), from fully expanded leaves. **K** and **L**: Stomata in the adaxial surface of WT leaves with paired subsidiary cells (K), whereas in *dil*338 the stomata sometimes had single subsidiary cells (arrows) (L).

Leaf (V3 and post-flowering stages) and stalk (post-flowering stage) sections were examined with a multiphoton laser scanning microscope (LSM) to determine the cytological variations in *dil1* mutants. The leaf epidermal cells of *dil1* mutant plants are irregular in size and shape, and are arranged more randomly when compared to those of the wild type at V3 (Figure 2A and 2B) and post-flowering stages (Figure 2C and 2D). The reduced length/width ratio in leaf epidermal cells may result in shorter and wider leaves in mutant plants. The reduced cell expansion along the longitudinal dimension may cause the cells to expand towards the adaxial or abaxial surfaces of the leaves, which results in bulging cells and wrinkled leaf surfaces. Stalk parenchyma cells in *dil1* mutants at the post-flowering stage are irregular in shape and distribution, and are significantly smaller than those of the wild-type plants (Figure 2E, 2F, 2G and 2H). The smaller cell size at least partially explains the shorter internodes in mutant plants.

In addition to the observed cell size and shape defects, defects in patterning of specific cell types on the adaxial surface of leaves were observed using a scanning electron microscope (SEM). In particular, the spacing of macrohairs was irregular, with about half of the macrohairs being clustered (Figure 2I and 2J). In addition, stomatal defects, where approximately 10% of stomata had only a single subsidiary cell, compared to the normal paired subsidiary cells seen in wild type, were observed (Figure 2K and 2L).

### Fine Mapping of *dil1*

A positional cloning approach was used to clone *dil1.* Two F_2_ populations (F_2_-*dil*338 and F_2_-*dil*474) were generated by crossing homozygous mutant plants with the maize inbred line A632. The segregation patterns in the F_2_ populations indicate that the mutant phenotypes were caused by recessive mutations at a single locus (Table S2). With 81 polymorphic SNP markers across the maize genome and 45 F_2_-*dil*338 and 53 F_2_-*dil*474 mutant plants, both mutants were mapped to the same interval on chromosome 6, between PHM788 (278 cM of IBM2 Neighbors map) and PHM1147 (394 cM of IBM2 Neighbors map).

**Figure 3:**
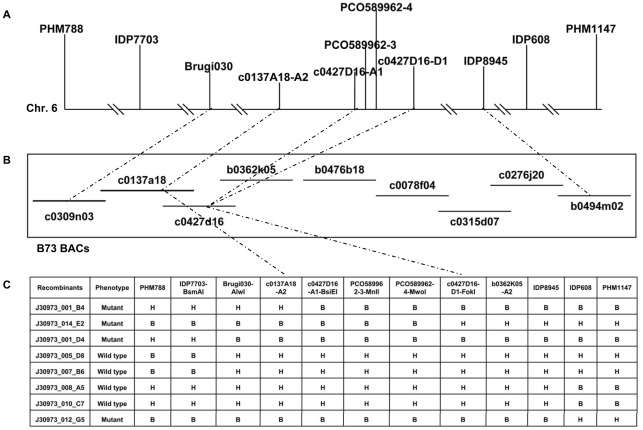
Fine mapping and cloning of *dil1*. **A**. DIL1 was mapped on the long arm of chromosome 6, between Brugi030 and IDP8945. **B**. B73 BAC contig covering the DIL1 locus. **C**. Recombination events near DIL1 delimited DIL1 between c0137A18-A2 and c0427D16-D1. A: wild type (A632) genotype. B: mutant (PHN4611) genotype. H: heterozygous genotype.

IDP and CAPS markers polymorphic between PHM788 and PHM1147 were used on 259 individuals from F_2_-*dil*474 and 275 individuals from F_2_-*dil*338 to further define the DIL1 locus. Both *dil*338 and *dil*474 mutants were mapped in the same interval between IDP7703 (285.9 cM) and IDP608 (316.8 cM) (Figure 3A), implying that *dil*474 and *dil*338 are allelic. To test whether *dil*338 and *dil*474 are allelic, individual F_2_-*dil*474 and F_2_-*dil*338 plants that were heterozygous at the DIL1 locus (as determined by two flanking markers) were reciprocally crossed and five F_1_ ears were generated. Mutant phenotypes were observed in F_2_ progenies from all five ears and the ratio between wild type and mutant plants was approximately 3∶1 (data not shown). It was therefore concluded that *dil*474 and *dil*338 are two alleles of the same gene and that mutations in this gene are responsible for the observed mutant phenotypes.

To further fine map and clone DIL1, 2484 F_2_ plants from *dil*338 were screened with flanking markers PHM788 and PHM1147 (Figure 3A), and 1240 recombinants were identified. DIL1 was further delimited to the interval between Brugi030 and IDP8945, spanning nine overlapping sequenced BACs (Figure 3B). Additional markers were developed from the BAC sequences (Table S1), and DIL1 was finally mapped between c0137A18-A2 and c0427D16-D1, spanning 214 kb (Figure 3C).

### Identification and Validation of Candidate Gene for *dil1*

**Figure 4:**
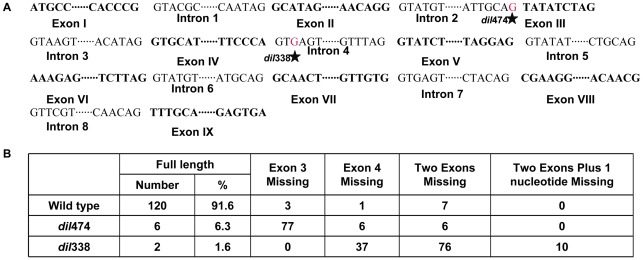
Single base pair mutations in *dil1* mutants cause alternative splicing. **A**. Structure of DIL1 gene and positions of single base pair mutations in *dil*338 and *dil*474 (in red and labeled with asterisk). **B**. Proportion of transcript variants in wild type and *dil1* mutants.

The closest flanking markers that define the DIL1 interval consist of two overlapping B73 BAC clones: c0137A18 and c0427D16. Only one low copy annotated gene with corresponding EST, an AP2 transcription factor-like gene, was identified within this interval (GeneBank EU966890.1). Three markers (c0427D16-A1, PCO589962-3 and PCO589962-4) developed from the AP2-like gene co-segregated with the phenotypes (Figure 3C). 5′ and 3′ RACE as well as RT-PCR were performed to generate the full length cDNA for the AP2-like gene and to determine its structure. The gene consists of nine exons and eight introns (Figure 4A, Figure S1), encoding a putative polypeptide of 412 amino acids. Sequence comparison of the AP2-like gene between PHN4611 and *dil*338 mutant revealed a point mutation (G to A) in the 4^th^ intron, near an exon-intron junction (Figure 4A). Interestingly, a point mutation (G to A) in the 2^nd^ intron, near another exon-intron junction, was detected in *dil*474 (Figure 4A). Identification of mutations within the same candidate gene in two independent mutant alleles confirms that DIL1 encodes the AP2 transcription factor-like gene.

Since both mutations occur near exon-intron junctions, RT-PCR with primers amplifying the full-length coding sequence was performed to determine the presence of splicing variants in *dil1* mutants. The cDNA products were cloned and sequenced. Sequences from 125 cDNA clones from *dil*338 and 95 cDNA clones from *dil*474 showed that mis-spliced variants represent the predominant form in both mutants (98.4% and 90.8% in *dil*338 and *dil*474, respectively) (Figure 4B). The predominant splicing variant in *dil*474 (deletion of exon 3) results in a 3-amino acid deletion, while the major splicing variant in *dil*338 (deletion of both exons 3 and 4) causes amino acid deletions and introduces a premature stop codon. Since both exons 3 and 4 are within the AP2 domain, deletions of exons 3 and/or 4 would presumably affect the function of the AP2 protein. It is interesting to notice that a small percentage (∼8%) of the AP2 transcripts in wild type plants also misses one or two of the exons, whereas a small percentage (∼2–6%) of the transcript is full length in *dil1* mutant plants. This indicates that the control of alternative splicing for this AP2 gene is not tight. Moreover, the mutations may not cause the complete loss of function for the AP2 gene, as a small number of functional transcripts are present in mutant plants.

### Expression of DIL1

**Figure 5:**
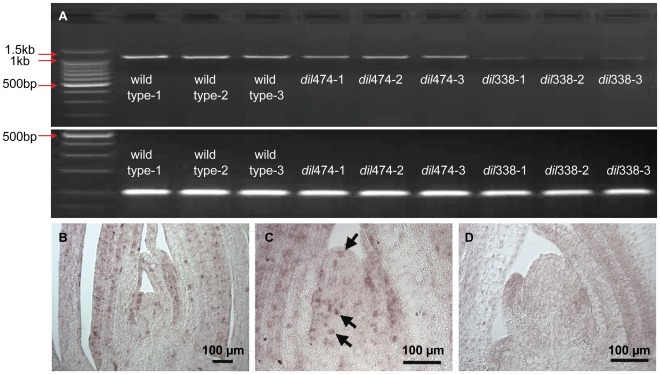
Expression analysis of DIL1 gene. **A**. DIL1 mRNA levels in V3 leaves in wild type and mutant plants, as determined by RT-PCR with 30 cycles for DIL1 (Upper), with the actin gene as the control (Lower). **B-D**. RNA in situ hybridization on tissue sections from A619 wild type vegetative apices, showing expression of DIL1 in the leaf primordia and the P_0_ region of the meristem (B and C). Hybridization signal was not observed with control, sense probes (D).

Besides the allelic variations at the amino acid level, the mutations may also affect transcript levels. Since the major splicing variant in *dil*338 contains a premature termination stop codon (PTC), it is likely to be targeted by the nonsense mediated mRNA decay (NMD) surveillance pathway. NMD is a quality-control mechanism that selectively degrades mRNAs harboring PTCs [14]. The expression levels of DIL1 in leaves between wild type and mutant plants were compared with semi-quantitative RT-PCR. The expression level of DIL1 gene in *dil*474 is similar to that of wild type plants, whereas the expression level in *dil*338 is significantly lower (Figure 5A). In comparison with *dil*474 transcript (with a nine nucleotide deletion), the larger deletion and in particularly the premature stop codon in *dil*338 transcripts may reduce the transcript stability by NMD, which results in the reduced steady-state level of the transcript.

A search of the DuPont/Pioneer MPSS database, which consists of libraries of 17-bp sequence tags from 2×10^5^ to 2×10^6^ cDNAs isolated from over 240 diverse maize tissues and developmental stages [15], with DIL1 indicates this gene is constitutively expressed in multiple tissues and developmental stages at a low level (data not shown). To further elucidate the expression patterns of DIL1, in situ hybridization was performed on tissue sections from A619 wild type vegetative apices (Figure 5B-D). Several independent hybridizations reveal that DIL1 transcripts accumulate in the leaf primordia and also the P_0_ region of the meristem, suggesting that DIL1 might control leaf shape from the early leaf initiation stage. Interestingly, the expression of DIL1 was not observed in all cells, but was in a subset of cells in a speckled pattern, suggesting that it might cell cycle regulated (arrowed, Fig. 5C). Hybridization signal was not observed with control, sense probes (Fig. 5D).

## Discussion

We identified two allelic maize mutants with semi-dwarf stature, wrinkled leaves, changed leaf shape and angles. The short statue of *dil1* mutant plants is due to decreased internode length, which is likely the result of smaller size and aberrant shape of the stalk parenchyma cells. The *dil1* mutant plants have wider but shorter leaves, which may be caused by the lower length/width ratio of the leaf epidermal cells. The reduced cell expansion along the longitudinal dimension may also cause the cells to expand toward the adaxial or abaxial surfaces of the leaves and give rise to the wrinkled leaf surfaces. In addition, defects in patterning of specific leaf cell types were observed, suggesting that *dil1* might function in control of intercellular signaling pathways required for cell specification and/or spacing.

### Mutations in an AP2 Transcription Factor-like Gene Affect Stalk and Leaf Development in Maize

A candidate gene for *dil1* was identified via positional cloning. It encodes an AP2 transcription factor-like gene. The presence of mutations within the same gene in two independently isolated mutant alleles confirmed the identification of the causal gene. AP2 genes belong to a large gene family of plant transcription factors, which contain the highly conserved AP2/ERF DNA binding domain and play important roles in developmental regulation as well as in response to abiotic and biotic stresses [16], [17]. Based on the number of AP2/ERF domains and the gene structure, the *AP2/EREBP* gene family has been divided into four subfamilies: AP2, RAV (related to ABI3/VP1), dehydration-responsive element-binding protein (DREB) and ERF [17], [18], [19]. A comprehensive phylogenetic analysis of 167 AP2-like genes in maize has been conducted recently ([17]), and DIL1 belongs to the AP2 subfamily (identical to ZmAP2-5 or Unigene ZM.83210 in [17]). While many genes of the RAV, DREB, and ERF subfamilies are involved in plant response to stresses, members of the AP2 subfamily have been implicated in developmental regulation. Genes from the AP2 subfamilies in Arabidopsis include AINTEGUMENTA (ANT), PLETHORA1 (PLT1) and WRINKLED1 (WRI1), all of which are involved in developmental regulation [20], [21], [22]. *Wri*1 mutants have wrinkled seeds due to altered fatty acid metabolism, but do not affect plant height or leaf development. The WRI1 protein binds to the AW-box sequence conserved among proximal upstream regions of genes involved in fatty acid biosynthesis [23]. AP2 transcription factors controlling meristem fates and inflorescence architecture in maize have been identified. Spikelet meristem (SM) identity is controlled by BD1 (*branched silkless1*), a putative AP2 transcription factor in the ERF subfamily, in maize [24]. *bd1* mutants initiate extra spikelets in the tassel, and in the ear, SMs are replaced with branch meristems. Two related AP2 transcription factors in the AP2 subfamily, *indeterminate spikelet1* (*ids1*) and *sister of ids1* (*sid1*), regulate the SM determinacy [25]. Double mutants of *ids1* and *sid1* do not make floral meristems (FMs). Instead, they initiate many bract-like organs. Expression patterns of meristem markers suggest that the SM never transitions to FM fate in the double mutants. It is of interest that a MIR172 family member (TS4) can negatively regulate both IDS1 and SID1 [25], [26]. Up-regulation of IDS1 results in extra florets, and sexual identity in maize is acquired by limiting floral growth through negative regulation of the floral homeotic pathway [26]. This observation indicates that the sex determination and SM determinacy pathways intersect, and AP2 transcription factors are involved in the regulation. Although the expression of certain AP2-like genes is regulated by microRNA, we failed to identify any putative microRNA binding sites in DIL1 (data not shown).

Although virtually all the genes cloned thus far from maize dwarf mutants are hormone-related, over-expression of several AP2 transcription factors produces dwarf phenotypes in Arabidopsis [27], [28], [29]. Also, several genes affecting cell shape have been isolated in maize and Arabidopsis, all of them are involved in cytoskeleton organization [30], [31], [32]. These observations indicate that DIL1 provides a new insight into regulation of plant architecture and cell specification. The phenotypic similarity between *dil1* mutants and mutants in the cytoskeleton may suggest that the genes identified in the cytoskeleton mutants are putative targets for DIL1.

The molecular mechanism of DIL1 regulation in stalk and leaf development is unknown. It could affect the expression of genes related to hormonal pathways (plant height) or cytoskeleton organization (leaf development). Thousands of differentially expressed genes in leaves and stalks between *dil1* mutant and wild type plants were identified in a microarray experiment (data not shown). Although many of the differentially expressed genes are involved in hormone metabolism or are hormone-responsive, it is virtually impossible to identify the genes that are directly regulated by DIL1 using this approach. A CHIP-seq (chromatin immunoprecipitation sequencing, [33]) experiment would be an appropriate approach to identify directly regulated genes, and may shed light on the molecular mechanism of DIL1 regulation in plant development. Alternatively, genetic screening to identify suppressors or modifiers (mutagenized or native alleles) for *dil1* would be a powerful way to identify genes that interact directly or indirectly with DIL1 [34], [35].

### Both *dil1* Alleles Result in Alternative Splicing Variants of the DIL1 Gene

Mutations in both *dil1* alleles occur in introns, near the exon-intron junctions. Since changes in introns would not directly affect amino acid sequences, one obvious possibility is their effect on intron splicing. Indeed, the mutations cause skipping of one or two exons, which results in the deletion of amino acids and introduction of premature stop codon. It is interesting to note that the aberrant splicing variants are present in wild-type plants and the full-length mRNA present in mutant plants, although at low frequencies. Mutations in introns cause a major shift in the ratio of full-length mRNA to the exon-skipping variants. Because the two exons skipped are located in the AP2 domain, the predominant splicing variants in *dil1* mutants presumably would not function properly. In addition, the dominant splicing variant in *dil*338 contains a premature termination stop codon (PTC), which could be targeted by the nonsense mediated mRNA decay (NMD) surveillance pathway [36], [37]. Indeed, the expression level of DIL1 is significantly lower in *dil*338 than that in *dil*474 or wild type plants.

Alternative splicing (AS) of pre-mRNA was first described more than 30 years ago [38]. Although it was once considered unusual, recent studies with high throughput sequencing indicate that most intron-containing human pre-mRNAs are processed to yield multiple mRNAs [39]. Alternative splicing is considered one of the main sources of proteomic diversity in multicellular eukaryotes [39]. In Arabidopsis and rice, a significant proportion of intron-containing genes are also alternatively spliced [36], [37]. Alternative splicing could be regulated developmentally and by environmental stimuli, resulting in functional consequences [40]. While exon skipping is the most prevalent form of alternative splicing in human [41], intron retention seems to be more common in plants [36], [37]. Alternative splicing is primarily regulated by RNA-binding proteins, which bind pre-mRNA near splicing sites and modulate the efficiency of their recognition by the basal splicing machinery or splicesome [40]. It would be of interest to investigate in detail how the specific point mutations in *dil1* mutants affect the splicing patterns.

The AS variants of DIL1 are from induced mutagenesis. Since AS is a ubiquitous phenomenon, it is possible that natural variations in AS are present in members of AP2 transcription factors. Indeed, the presence of AS variants of certain AP2-like genes have been reported in barley[42], wheat [43], maize [44] and kiwifruit [45]. The AS patterns in the homologous barley and wheat AP2 gene are conserved and differentially regulated by various abiotic stresses, implying a regulatory role at the level of AS in response to abiotic stresses. ZmDREB2A, a DREB2 homolog in maize, produces two forms of transcripts and only one of them is functional [44]. Under normal conditions the “defect” isoform is present but the functional isoform is undetectable. However, the functional variant is much more responsive to stress treatments. The data indicate that AS plays an important role in regulating ZmDREB2A activity under stress conditions. Thus, AS variants are not only present in AP2 genes, but AS regulation may have important implications for stress responses. It is possible that AS regulation on the AP2 transcription factors is ubiquitous. With the advance of Next Gene Sequence technologies, it becomes increasingly feasible to identify and characterize AS variants for all the AP2 genes.

### Regulation of Plant Stature and its Application

The genetic control of plant architecture is very complex [2]. Reduced plant height and leaf angles are desirable traits in maize breeding [1]. Such plants provide better tolerance to lodging and are more adaptable to high planting density. Mutations in DIL1 reduce internode length and leaf angle, but also cause undesirable pleiotropic effect (e.g. wrinkled leaves). Tightly controlled transgenic expression of DIL1 with tissue-specific promoters may be a way to generate the desirable phenotypes without the negative pleiotropic effect (e.g. [46]). An alternative approach would involve searching for naturally occurring alleles of DIL1 with preferred phenotypes. A third approach is to identify genes which act downstream of DIL1 through genetic screening [34], [35]. Such genes may give more specific phenotypes that can be used for crop plant improvement.

## Materials and Methods

### EMS Mutagenesis for Dwarf Mutant Screening

An ethyl methanesulfonate (EMS) mutagenized population was generated in PHN4611, an elite, Non-Stiff Stalk (NSS) inbred lines from Pioneer Hi-bred International, Inc. Fresh PHN4611 pollen was suspended in a 0.06% solution of EMS in paraffin oil (Fisher 0121-1) for 45 min with shaking at 5 min intervals. A ratio of ten parts EMS solution to one part pollen was used. The EMS treated pollen was then applied to silks of the PHN4611 with a camel hair brush. The M1 individuals were self-pollinated to generate the F_2_s families, which were screened for alterations in plant and organ growth. Approximately 500 M2 families of the EMS mutagenized maize populations were grown in the greenhouse in 18-plant flats and screened. The number of plants per family grown varied and depended upon the seed availability. Seedling plant architecture characteristics such as, but not limited to, leaf initiation rate, leaf morphology, seedling size, leaf angle, leaf length, leaf width of mutant plants and wild type plants were observed at different stages during the germination and seedling growth.

Phenotypic changes were identified and monitored. At approximately the three leaf stage, mutant phenotypes became obvious and distinct from the wild type. Mutants that fit approximately the segregation ratio of 3∶1 were identified as recessive mutations and advanced for further characterization.

### Cytological Analysis of Mutant Leaf Epidermal Cells and Stalk Parenchyma Cells

Leaf samples were collected by cutting a 2 cm wide strip from the mid-point of the 2^nd^ leaf of V3 seedlings (3–4 week old plants, when the collar of the 3^rd^ leaf is visible) and from the first leaf below the ear of mature plants. Samples were fixed in a solution of 25% acetic acid and 75% ethanol. Samples were further processed by taking 6 mm leaf punches from corresponding regions of fixed tissue and post-fixed in 2% glutaraldehyde to enhance host cell autofluorescence. These post-fixed leaf disks were rinsed, cleared in chloral hydrate, mounted in Hoyer’s medium, and examined with the 720 nm laser line of a Zeiss multiphoton laser scanning microscope (LSM), using a 20x Plan Apochromat (0.75 NA) objective lens. Multiple optical sections (0.8 µm section thickness) were collected and maximum intensity projections assembled as single images for evaluation.

Maize stalk samples were collected by cutting 2 cm wide cross-sections of stalk from the center of internode #3 (base) and #9 (apex) and were fixed in acetic acid-ethanol. Cross-sections were post-fixed in glutaraldehyde to enhance cell wall autofluorescence, cleared in chloral hydrate, mounted and examined with multiphoton LSM.

*dil1* mutants and wild type sibs were grown for ∼5 weeks in the growth chamber, and leaf tissue from the base of the blade was dissected from leaves that were emerging from the whorl or from leaves that were almost fully expanded. Leaf pieces were mounted on aluminum stubs using silver paint (Electron Microscopy Sciences, Hatfield, PA, USA), then imaged in a Hitachi S3500N scanning electron microscope under high vacuum mode, and 5keV accelerating voltage, as described in Jackson (2002)[47].

### Fine-mapping and Cloning of DIL1

Two large F_2_ populations (F_2_-*dil*338 and F_2_-*dil*474) were generated from homozygous *dil* mutant plants and A632. For initial chromosomal localization of the mutants, 45 F_2_-*dil*338 and 53 F_2_-*dil*474 mutant plants were genotyped with 81 SNPs distributed across the maize genome. Additional IDP and SNP markers around the DIL1 locus were developed with publicly available IDP and BAC sequences. In the end, the DIL1 interval was delimited into a two overlapping BAC interval and a single candidate gene was identified.

### RT-PCR and RACE

Total RNA was extracted from the leaves of wild type and homozygous mutant plants using a Qiagen RNeasy kit (Qiagen Inc., Valencia, CA, USA) and cDNA obtained with oligo DT and Superscript® reverse transcriptase (Invitrogen, Carsbad, CA, USA). PCR was performed using GoGreen mixture (Promega, Madison, WI, USA). Primers for DIL1 are 5′-CTCGTCCTCGTCGCTCAG-3′ and 5′-AACCGTTGTTGTTGTTATTGTCG-3′, and primers for Actin are 5′-CACTGTGCCAATCTACGAGGGT-3′ and 5′-CACAAACGAGGGCTGGAACAAG-3′. RT-PCR products were fractionated in a 1.5% agarose gel and visualized by ethidium bromide staining.

5′ and 3′ RACE were carried out with the GeneRacer™ kit (Invitrogen). Total RNA was extracted from maize tissue using Trizol reagent (Invitrogen) and treated with RNase-free DNase I (Promega). Additional steps include dephosphorylation and decamping of mRNA, ligating the GeneRacerTM RNA oligo to full-length mRNA, reverse transcription with RT and GeneRacerTM oligo dT primers, performing PCR with the GeneRacerTM primer and gene specific primers, gel imaging, cloning and sequencing PCR products, and analyzing sequencing results. The primers used include 5′-GCTGTCAACGATACGCTACGTAACGGCATGACAGTG (T) -3′ for reverse transcription, 5′-CGACTGGAGCACGAGGACACTGA-3′ and 5′-CGACCTGTCCACCTATGCCGGGTGAC-3′ for 5′RACE first round amplification, 5′-GGACACTGACATGGACTGAAGGAGTA-3′ and 5′-AACGCCCTCCTGGGCACCAGCTC-3′ for 5′RACE nested PCR amplification, 5′-GCTGTCAACGATACGCTACGTAACC-3′ and 5′-TGGCTTCAGTTCTCCATCAAAGCCCTCT-3′ for 3′RACE first round amplification, 5′-CGCTACGTAACGGCATGACAGTG-3′ and 5′-GAGTACCTGGAGCCCTGCTGATCCTTCC-3′ for 3′RACE nested PCR amplification.

### RNA *in Situ* Hybridization

Sample preparations and *in situ* hybridizations were performed as described previously [48], except that 8% polyvinyl alcohol (MW 70.000 – 100.000, SigmaP1763) was included in the staining buffer. The DIL1 probe contained the nucleotide sequences for about 600 bp coding region and 130 bp 5′UTR. The sequences were cloned into pCR-Blunt II-TOPO vector in sense and antisense orientation, relative to the T7 promoter. Linearized plasmids were used as templates for probe synthesis using T7 RNA polymerase. Probes were not hydrolyzed. After the color reaction, slides were mounted in 30% glycerol and photographed using DIC microscopy.

## Supporting Information

Figure S1 **Genomic sequence of DIL1. Exons are labeled in bold.**
(TIF)

Table S1 **Primer and restriction enzyme info of markers near the DIL1 locus.**
(TIF)

Table S2 **X^2^ test on the segregation patterns in the F_2_-** ***dil*** **338 and F_2_-** ***dil*** **474 populations.**
(TIF)
