# Supplementary figures and images for: Mutations in an AP2 Transcription Factor-Like Gene Affect Internode Length and Leaf Shape in Maize
Source: PLoS One. 2012 May 23;7(5):e37040. doi: 10.1371/journal.pone.0037040 (PMC3359370; doi:10.1371/journal.pone.0037040)

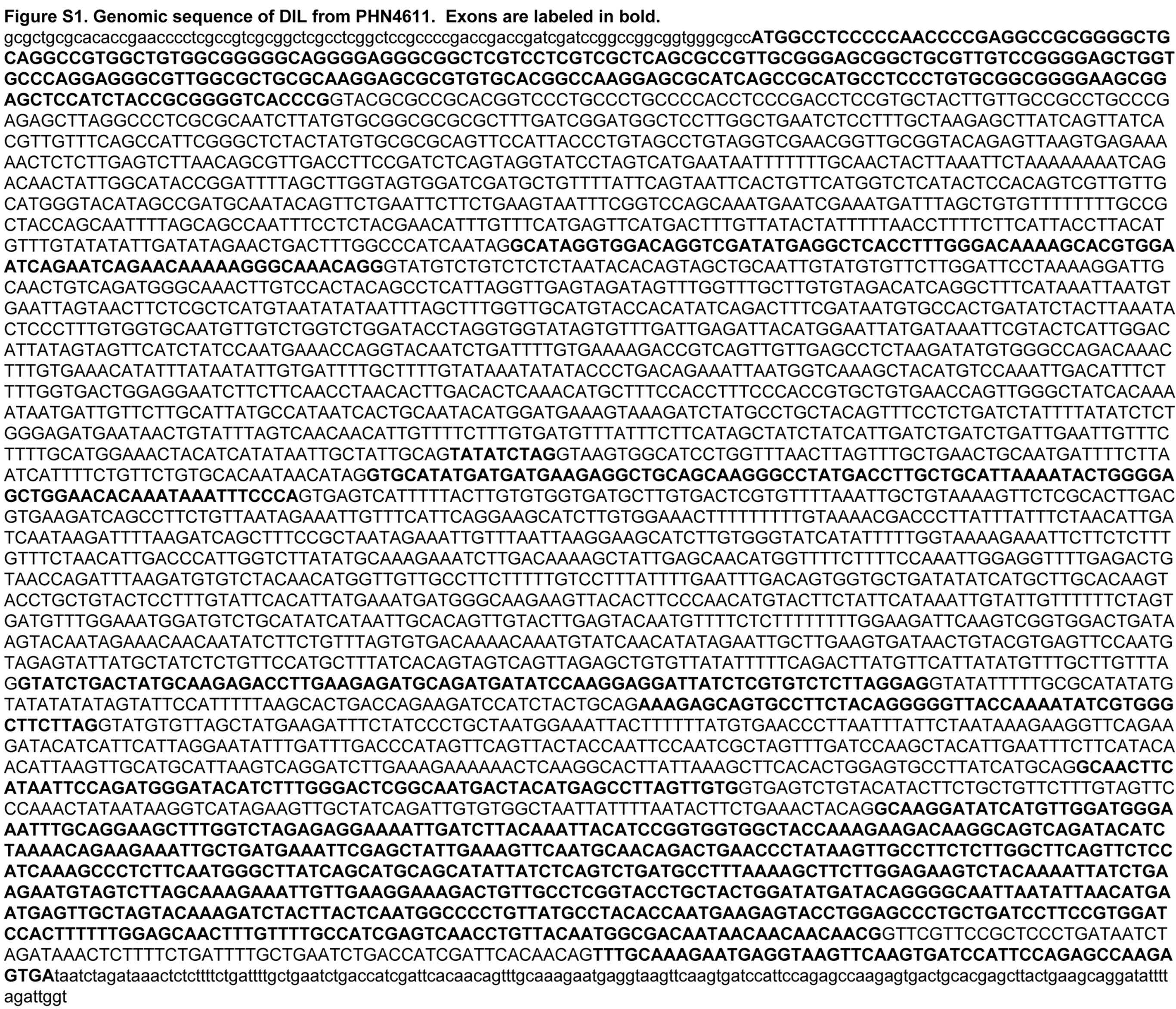

Supplement: Figure S1 — Genomic sequence of DIL1. Exons are labeled in bold. (TIF) [file pone.0037040.s001.tif]

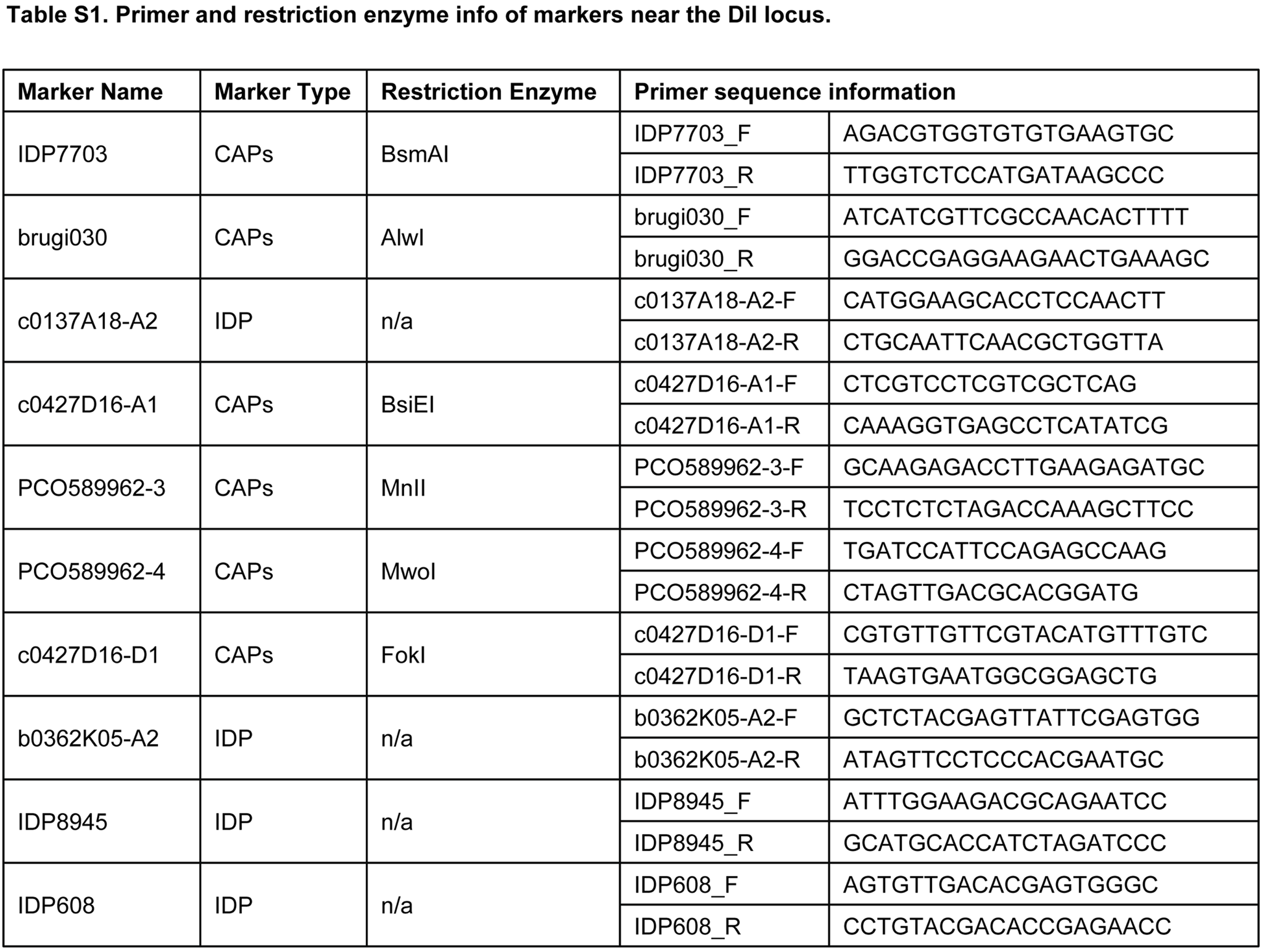

Supplement: Table S1 — Primer and restriction enzyme info of markers near the DIL1 locus. (TIF) [file pone.0037040.s002.tif]

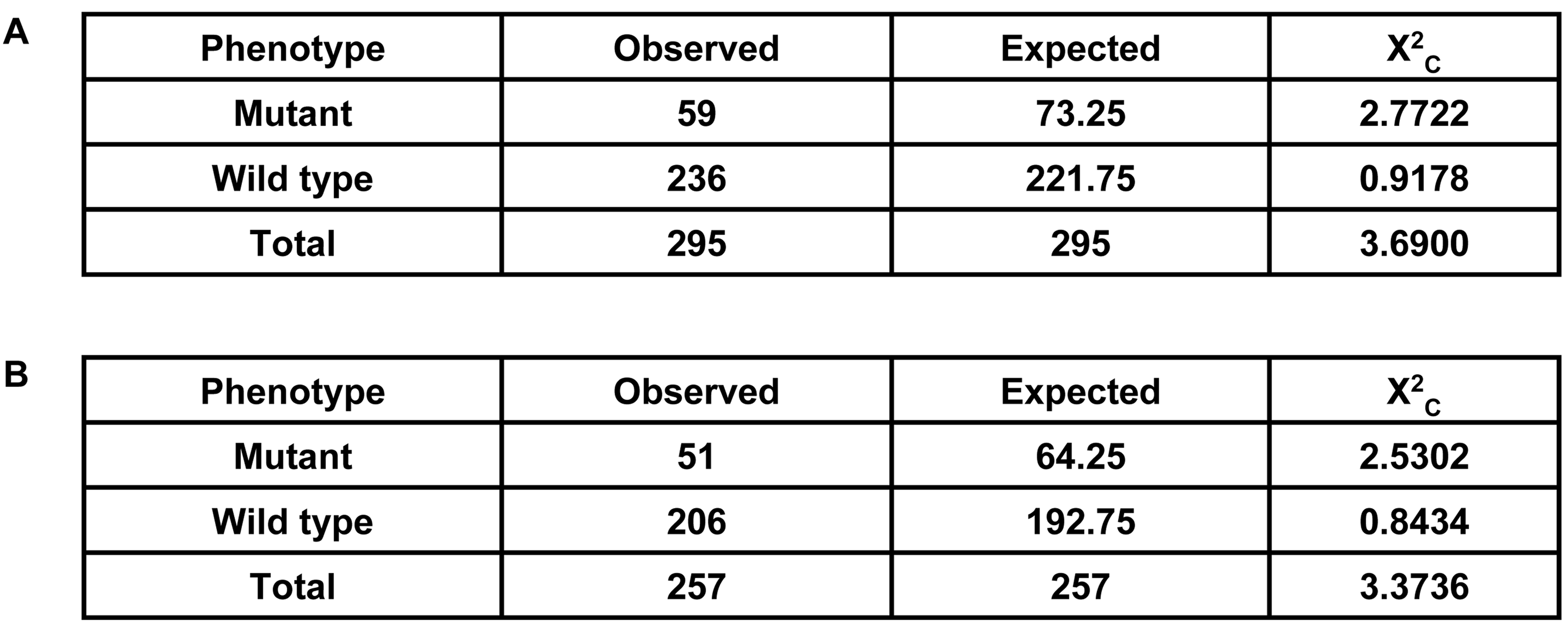

Supplement: Table S2 — X2 test on the segregation patterns in the F2- dil 338 and F2- dil 474 populations. (TIF) [file pone.0037040.s003.tif]
